# Editorial: Lipidomics of oxylipins in biological systems

**DOI:** 10.3389/fphar.2023.1342596

**Published:** 2023-12-06

**Authors:** Paola Patrignani, Nils Helge Schebb, Oliver Werz, Dieter Steinhilber

**Affiliations:** ^1^ Paola Patrignani, “G. d’Annunzio” University, Chieti, Italy; ^2^ Nils Helge Schebb, University of Wuppertal, Wuppertal, Germany; ^3^ Oliver Werz, Friedrich Schiller University Jena, Jena, Germany; ^4^ Dieter Steinhilber, Goethe University, Frankfurt, Germany

**Keywords:** lipid mediators, lipidomics, oxylipins, eicosanoids, prostanoids, leukotriene

Polyunsaturated fatty acids (PUFAs) have many oxidizable double bonds, making them susceptible to enzymatic and free radical oxidation. These include arachidonic acid (AA), eicosapentaenoic acid (EPA), and docosahexaenoic acid (DHA). Oxygenated lipids are collectively known as oxylipins. Eicosanoids are a group of oxylipins derived from arachidonic acid (AA) and are biologically important to mammals. Prostanoids and leukotrienes (LTs) are the most studied examples of eicosanoids. These biologically active lipids play a role in various pathological processes, including inflammation, cancer, and atherothrombosis. The interest in eicosanoids stems from their connection to important pharmacological classes, such as non-steroidal anti-inflammatory drugs (NSAIDs), which relieve pain and stiffness in various acute and chronic musculoskeletal disorders, and anti-LT drugs used to treat asthma. These drugs affect oxylipin biosynthesis and responses, making eicosanoids a crucial area of research. The process of oxygenation of eicosanoids involves several enzymatic steps. It starts with the release of PUFAs from membrane phospholipids by phospholipases. The final biosynthetic steps of these biologically active metabolites are carried out by primary oxidative enzymes such as cyclooxygenases (COXs) and lipoxygenases (LOXs), along with isomerases, synthases, and hydrolases, all working in a coordinated manner. Aspirin is an NSAID that irreversibly affects COX-1 and COX-2 activity via the acetylation of Serine529 and Serine516, respectively, in the cyclooxygenase active site. It is an antiplatelet agent used at low doses to prevent cardiovascular diseases (Patrignani and Patrono, 2015). The main product of COXs from AA is prostaglandin (PG)H_2_. Small amounts of 11- and 15-hydroperoxyeicosatetraenoic acid (HpETE), subsequently converted to the corresponding hydroxyeicosatetraenoic acids (HETEs), are produced in the presence of high concentrations of AA. It has been suggested that acetylated COX-2 can produce enhanced levels of 15R-HETE responsible for forming the “aspirin-triggered lipoxin (LX)" 15-epi-LXA_4_. However, there is still a debate regarding this issue. The 18R-HEPE (18R-hydroxyEPA) and 17-HDHA (17-hydroxyDHA) are reported to be formed via aspirin-acetylated COX-2 from EPA and DHA, respectively. *In vitro* studies show that they can only be formed at low rates compared to PGH_2_ by native COX-2 (Sharma et al., 2010).

Oxylipins also include trihydroxylated, AA-derived lipoxins, and di- or trihydroxylated derivatives of EPA and DHA; some are termed “specialized pro-resolving mediators” (SPMs) that have been proposed as mediators of inflammation resolution. Families of SPMs described in the literature include lipoxins, resolvins, maresins, protectins, and their peptide conjugates. Formation of lipoxins or resolvins usually involves AA 5-lipoxygenase (5-LO, ALOX5) and different types of AA 12- and 15-lipoxygenating paralogues (15-LO1, ALOX15; 15-LO2, ALOX15B; 12-LO, ALOX12). Thus, depending on the sequence of action, lipoxins and resolvins can be formed via the 5-LO:12/15-LO pathway or the 12/15-LO:5-LO pathway. The dihydroxylated SPMs, namely, protectins and maresins, can be formed by 15-LO1/2 alone. However, the initial product of 15-LO, 17S-hydroperoxy-DHA can be converted enzymatically first to a 16S, 17S-epoxide and then to protectin D1 by an appropriate epoxide hydrolase.

One hallmark of SPM formation is that the reported levels of these lipid mediators are much lower than typical pro-inflammatory mediators, including the monohydroxylated fatty acid derivatives (e.g., 5-HETE), LTs, or certain COX-derived prostanoids. Thus, reliable detection and quantification of these metabolites is challenging, and a recent article shows that inadequate methods have been used incorrectly, demonstrating the presence of SPMs in biological samples (O´Donnell et al., 2023). Based on that and the unclear formation and signaling, the endogenous role of some of the SPMs has recently been questioned (Schebb et al., 2022).

The production of prostanoids, LTs, and SPMs occurs in single cells or in cell mixtures by transcellular biosynthesis through the cooperation of multiple different types of cells in the tissues.

In addition, cytochrome P450 (CYP) enzymes, a superfamily of heme-containing monooxygenases, catalyze hydroxylation and epoxidation of PUFAs such as AA yielding 20-hydroxyeicosatetraenoic acid (20-HETE) and epoxyeicosatrienoic acids (EETs); both are implicated in blood pressure regulation and inflammation. The enzyme soluble epoxide hydrolase (sEH) converts EETs to less bioactive dihydroxyeicosatrienoic acids (DHETs). EETs exhibit anti-inflammatory, analgesic, antihypertensive, cardio-protective, and organ-protective properties. sEH inhibitors maintain endogenous EET levels and reduce DHET levels, resulting in therapeutic potentials for cardiovascular, central nervous system, and metabolic diseases.

Oxylipins can be covalently bound in several lipids, such as oxidized phospholipids (oxPL), endocannabinoids, and cholesteryl esters (CE). Enzymatically oxidized phospholipids (eoxPL) are pro-coagulants and promote different actions in leukocytes, platelets, and cancer cells.

This Research Topic includes six research papers. Bergqvist et al. used mass spectrometry to compare the effects of the mPGES-1 inhibitor (the enzyme downstream of COX-2 involved in the biosynthesis of PGE_2_) Compound III (CIII) (LeClerc et al., 2013) with the cyclooxygenase (COX)-2 inhibitor NS-398 on protein and lipid profiles in interleukin (IL)-1β-induced A549 lung cancer cells. CIII reduced proliferation and enhanced the cytotoxicity of cisplatin, etoposide, and vincristine. Inhibiting mPGES-1 or COX-2 resulted in different protein and lipid profiles. The results have important implications for using mPGES-1 inhibitors as a cancer treatment and should be verified in clinical studies. Di Francesco et al. characterized a new anti-inflammatory compound called AF3485, which inhibits human mPGES-1. They found that it can control COX-2 induction caused by inflammatory stimuli. The compound also induced endothelial COX-2-dependent PGI_2_ production via PPARγ activation, both *in vitro* and *in vivo*, which might translate into a protective effect for the cardiovascular system. Aspirin (acetylsalicylic acid, ASA) is recommended for the secondary prevention of atherothrombotic events and has shown anticancer effects. The current enteric-coated drug formulation may reduce aspirin bioavailability. Liquid formulations could improve aspirin pharmacokinetics and pharmacodynamics. IP 1867B is a liquid-aspirin formulation that combines three ingredients: ASA/triacetin/saccharin. Hofling et al. found that IP 1867B was more potent in affecting serum thromboxane (TX)B_2_ generation than ASA. TXB_2_ is a non-enzymatic metabolite of TXA_2_, a potent proaggregatory platelet agonist. The relevance of this finding deserves evaluation *in vivo* in humans. In cancer cells, ASA and IP 1867B acted by inhibiting PGE_2_ and TXB_2_ generation via the acetylation of COX-2. ASA and IP867B at clinically relevant concentrations did not substantially induce the biosynthesis of 15R-HETE and 15-epi-LXA_4_. The data do not support the relevance of the so-called aspirin-triggered SPM formation. Leineweber et al. explored the effects of sorafenib, a multikinase inhibitor approved for advanced hepatocellular carcinoma (HCC), on PUFA-derived epoxygenated metabolites. The drug is also a potent sEH inhibitor. While AA-derived EETs can promote tumor growth and metastasis, DHA-derived 19,20-epoxydocosapentaenoic acid (19,20-EDP) has anti-tumor activity in mice. Leineweber et al. found that sorafenib caused an increase in 19,20-EDP and its dihydroxy metabolite in 43 patients with HCC while decreasing DHA plasma levels. These findings suggest that taking DHA supplements could help to increase the levels of the anti-tumor compound 19,20-EDP in HCC patients undergoing sorafenib therapy. Dahlke et al. studied 5-LOX-activating protein (FLAP) antagonists. FLAP is known to provide AA as a substrate to 5-LOX for generating LTs. Screening of multiple prominent FLAP antagonists for their effects on lipid mediator formation in human M1-and M2-monocyte-derived macrophages showed that some compounds (especially those that contain an indole or benzimidazole moiety) reduced the production of 5-LOX-derived LTs but increased the formation of dihydroxy fatty acids belonging to the SPMs from DHA, namely, resolvin D5 and PDx. This confirms that mainly dihydroxylated SPMs such as RvD5 but not trihydroxylated SPMs are formed by human leukocytes. The authors concluded that the antagonism of FLAP suppresses the conversion of AA by 5-LOX to LTs and lipoxins but not the conversion of DHA to 7-HDHA and RvD5, which should be considered for developing such compounds as anti-inflammatory drugs. The data demonstrate that RvD5 formation via the 5-LO:15-LO pathway (where DHA is first oxidized by 5-LOX and then by 15-LOX) is FLAP-independent, whereas the conversion of AA and EPA by 5-LO requires FLAP. The research paper by Dahlke et al. complements previous findings that showed FLAP-dependency of RvD5 formation via the 12/15-LO:5-LO pathway where 15-LOX-derived 17-HDHA is then converted by 5-LOX to RvD5 (Mainka et al., 2022). Based on the current data, it can be concluded that RvD5 biosynthesis in M2-like macrophages occurs via the 5-LO:15-LO pathway and is FLAP-independent. Nagata et al. investigated the effects of the EPA metabolite (±)5 (6)-dihydroxy-8Z,11Z,14Z, 17Z-eicosatetraenoic acid [(±)5 (6)-DiHETE] on allergic conjunctivitis (AC) using a mouse model. They found that topical or intraperitoneal (±)5 (6)-DiHETE treatment broadly suppressed AC pathology and could be a novel treatment option for AC.
